# The Surface Area to Volume Ratio Changes the Pharmacokinetic and Pharmacodynamic Parameters in the Subcutaneous Tissue Cage Model: As Illustrated by Carprofen in Sheep

**DOI:** 10.3389/fvets.2022.905797

**Published:** 2022-07-01

**Authors:** Richard Munn, Ted Whittem, Andrew P. Woodward

**Affiliations:** ^1^Faculty of Veterinary and Agricultural Sciences, University of Melbourne, Werribee, VIC, Australia; ^2^Cognosco, Anexa, Morrinsville, New Zealand; ^3^Division of Tropical Health and Medicine, College of Public Health, Medical and Veterinary Sciences, James Cook University, Townsville, QLD, Australia; ^4^Faculty of Health, University of Canberra, Canberra, ACT, Australia

**Keywords:** pharmacokinetics, pharmacodynamic, surface area to volume ratio (SAV), tissue cage model, sheep, carprofen

## Abstract

**Introduction:**

Pharmacokinetic and pharmacodynamic models can be powerful tools for predicting outcomes. Many models are based on repetitive sampling of the vascular space, due to the simplicity of obtaining samples. As many drugs do not exert their effect in the vasculature, models have been developed to sample tissues outside the bloodstream. Tissue cages are hollow devices implanted subcutaneously, or elsewhere, that are filled with fluid allowing repetitive sampling to occur. The physical dimensions of the cage, namely, the diffusible surface area to volume ratio, would be expected to change the rate of drug movement into and out of tissue cages.

**Methods:**

Seven sheep were implanted with five pairs of tissue cages, subcutaneously. Each pair of cages had a different length but a fixed diffusible surface area, so the surface area to volume ratio differed. Carrageenan was injected into half of the cages in each animal during one sampling period in a cross-over design. Samples from each cage and the bloodstream were obtained at 14-time points during two sampling periods. The concentration of carprofen was measured using LC–MS/MS and the results were modeled using nonlinear mixed-effects techniques. Prostaglandin metabolites were also measured and the change over time was analyzed using linear mixed effect modeling.

**Results:**

The presence of carrageenan within an animal changed the systemic pharmacokinetics of carprofen. The rate of drug movement into and out of the tissue cages varied with the surface area to volume ratio. The concentration time curve for prostaglandin metabolites changed with cage size.

**Conclusion:**

The surface area volume ratio of tissue cages will influence the calculated pharmacokinetic parameters and may affect calculated pharmacodynamics, thus, it is an important factor to consider when using tissue cage data for dosing regimes.

## Introduction

Pharmacokinetic and pharmacodynamic (PKPD) models are powerful tools for predicting outcomes from pharmacological interventions when they accurately model reality. Many PKPD models are based on sampling from the vascular space as blood, plasma, and serum are easily sampled over multiple time points with reliable techniques. However, the vasculature is not the target site of action for many drugs, e.g., antibiotics and anti-inflammatories are commonly given to treat ailments outside the bloodstream. To improve the accuracy of PKPD model predictions, *in vivo* models have been developed to obtain and measure drug concentrations and effects in other tissues.

Ideally, these *in vivo* models allow individual tissues within an animal to be sampled with high frequency over a relatively short period of time. Clearly, tissue collection that requires the sacrifice of animals or produces significant damage to the tissue (e.g., muscle biopsy) is not ideal. Therefore, the tissue cage (or chamber) model, which was developed by Guyton (1) to study physiological parameters, was quickly adopted for pharmacological studies of antibiotics (2). Hollow devices (cages) are implanted subcutaneously or within tissue in a manner that allows percutaneous sampling by a needle and syringe. Cages are made permeable to drugs by perforating portions of their surfaces, thus, creating a diffusible surface area. Two to three weeks after implantation, cages are filled with a fluid that can be sampled (3, 4). This model has the advantage of producing relatively large sample volumes compared with alternative approaches that use skin windows, blistering, or wicking. Furthermore, the cages can be maintained for long periods (e.g., 22 weeks in cattle) and still produce viable samples (3).

Several variations in tissue cage shape, material, and size have been used in published studies. While silicone cylinders, as described by Bengtesson and Sidhu (3, 4), are the most common construction material and shape, the size of cages can vary significantly between studies. This variation in size, coupled with variations in the diffusible surface area, leads to variations in the surface area to volume ratio (SA/V) of the cages (Table 1). The SA/V parameter, under the Fick's law of diffusion, is expected to be an important contributor to the pharmacokinetics that are subsequently measured. Modeling of various SA/V ratios *in vitro* showed a marked difference in the pharmacokinetics of the cages according to their SA/V (5). An *in vivo* model by Van Etta also showed that cages of differing dimensions but the same SA/V produce the same pharmacokinetic profiles (6), consistent with the theoretical prediction under the Fick's law.

**Table 1:** Previously published papers using the tissue cage model with NSAIDs. Calculated SA/V from tissue cage descriptions.

**References**	**Drug**	**Species**	**SA:V**	**Carrageenan**
McKellar et al. (7)	Carprofen	Dog	-	
Caldwell et al. (8)	Diclofenac topically	Horse	0.49	Yes
Espinasse et al. (9)	Flunixin and Tolfenamic acid	Calves	0.55	
Pelligand et al. (10)	Robenacoxib	Cat	0.45	
Cheng et al. (11)	Phenylbutazone	Donkey		
Cheng et al. (12)	Phenylbutazone, Flunixin	Sheep	0.29	
Arifah et al. (13)	Ketoprofen	Goat	0.17	Yes
Landoni and Lees (14)	Ketoprofen	Horses	0.14	Yes
Landoni et al. (15)	Tolfenamic acid	Calves	0.14	Yes
Arifah et al. (16)	Ketoprofen	Sheep	0.17	
Landoni et al. (17)	Ketoprofen	Sheep	0.14	Yes
Landoni et al. (18)	Ketoprofen	Calves	0.14	Yes
Cheng et al. (19)	Carprofen	Sheep	0.28	
Lees et al. (20)	Carprofen	Horse	0.14	
Cheng et al. (21)	Carprofen	Sheep	0.28	Yes
Pelligand et al. (22)	Robenacoxib, Ketoprofen	Cat	0.45	Yes
Arifah and Lees (23)	Phenylbutazone	Calves	0.14	
Lees et al. (24)	Carprofen	Calves	-	Yes

*“-”insufficient information in the article to calculate*.

In addition to the variation in physical characteristics, inflammation may be induced using carrageenan within one or more cages when studying anti-inflammatory drugs. This is designed to allow comparison between inflamed spaces and non-inflamed spaces within an individual animal (12, 19, 25, 26). It is unknown if inducing inflammation within one or more cages in an animal changes the observations in either the blood or in the non-inflamed cages compared to similar observations with no inflammation.

The tissue cage model continues to be used for pharmacological research (27, 28) without standardization or comprehensive verification. Notably, the dosing interval for robenacoxib of 24 h is based on tissue cage modeling in cats (10).

This study aimed to evaluate the effects of SA/V and inflammation on the PK and PKPD results from tissue cage models, using the well-characterized NSAID drug carprofen. We predicted that as the volume of the cage increases relative to the surface area, changes in the pharmacokinetics will result in changes in the predicted pharmacodynamic parameters. Second, we predicted that the presence of carrageenan induced inflammation within the individual animal would change the pharmacokinetics of the non-inflamed cages.

## Materials and Methods

In this experiment, we simultaneously sampled blood and tissue cage fluid from implanted cages with varying SA/V and with and without induced inflammation, in a cross-over design.

Tissue cages were prepared in the manner of Sidhu et al. (4) utilizing 17 mm outer diameter silicone laboratory tubing. In total, five sizes were prepared; 3, 6, 10, 14, and 18 cm in length. Each end was sealed with silicone putty and 24 holes were created in each using a 4 mm biopsy punch. Once the putty had set, the cages were packaged in sets of five and sterilized by ethylene oxide. The calculated surface area, volume and SA/V ratio are displayed in Table 2.

**Table 2:** Dimensions of the subcutaneously implanted tissue cages.

**Length (cm)**	**Diffusable surface**	**Volume**	**Surface area to volume**
	**area (cm^**2**^)**	**(mL)**	**ratio (cm^**−1**^)**
3	3.0159	5.3014	0.5689
6	3.0159	10.6029	0.2844
10	3.0159	17.6715	0.1707
14	3.0159	24.7400	0.1219
18	3.0159	31.8086	0.0948

In total, seven merino wethers, approximately 18 months old and ranging from 39 to 59 kg, were enrolled (University of Melbourne Animal Ethics approval 1814590). Each wether was determined to be healthy by veterinary clinical examination and routine hematological and biochemical testing prior to enrolment. All the sheep were housed in a corrugated iron shed on slatted floors with water supplied *ad libitum*. Pellets (Sheep & Cattle Rumevite, Townsville, QLD, Australia) and lucerne chaff were provided daily. Ventilation was provided by passive air movement through doors and windows, and experiments were conducted between April 2019 and August 2019 in Werribee, Victoria, Australia (29).

To prepare the animals, under general anesthesia, five hollow silicone cylinders were implanted subcutaneously on each side of the neck of each wether to form ten tissue cages as described previously (29). The cages were inserted in size order, with the shortest cage being most cranially positioned.

A cross-over two-phase pharmacokinetic study was conducted at 3 and 7 weeks after implantation of the tissue cages. An indwelling over-the-needle intravenous catheter was placed in a cephalic or jugular vein (18 ga Jelco Optiva, Smiths Medical Macquarie Park, NSW) and an injection port was attached and flushed with heparinised saline between each use. At time zero, 4 mg/kg carprofen (Rimadyl LA, Zoetis Australia) was injected intravenously in the contralateral vein to the catheter.

During one of the phases, 1 ml of 1% κ-carrageenan was injected into the five cages on a single side (randomized between left and right) at time −2 h to induce mild inflammation. Tissue cage fluid was aspirated from each of the 10 tissue cages at times −1, 0.5, 1, 2, 3, 4, 5, 6, 8, 12, 24, 36, 48, and 72 h. These samples were obtained by inserting a 20 ga hypodermic needle through the skin directly into each tissue cage. Analgesia was provided by the Coolsense device (Coolsense, Balance Medical, Kenmore East, QLD). Details of the use of the Coolsense device have been reported elsewhere (29). At each time point, a blood sample was also obtained *via* the catheter or by direct venipuncture. Each set of 11 samples took approximately 15 min to collect. Tissue cage fluid was transferred into 1.3 ml lithium heparin tubes while blood samples were divided between lithium heparin tubes and serum tubes containing indomethacin (C_19_H_16_ClNO_4_, CAS: 53-86-1) to prevent *ex vivo* formation of eicosanoids (30). All the samples were kept at 4°C until centrifugation and decanting of plasma, serum, and the liquid fraction of tissue cage fluid into 1.5 ml microcentrifuge tubes and then stored at −80°C until analysis.

### Analytical Methods

#### Reagents

Deionized water was purified using a MilliQ system to 18 MΩ (Millipore North Ryde NSW). Chromatography grade acetonitrile (ACN) and formic acid were sourced from Merck Australia (North Ryde, NSW). Carprofen analytical standard (C_15_H_12_ClNO_2_, CAS: 53716-49-7) and meclofenamic acid (MFA) analytical standard (C_14_H_11_Cl_2_NO_2_, CAS: 644-62-2) were obtained from the Sigma–Aldrich Australia (North Ryde, NSW). High throughput 96 well protein precipitation and phospholipid removal plates (Ostro, Waters Australia Rydalmere, NSW) and 1 ml polypropylene round bottom 96 well plates were obtained from Waters Australia (Rydalmere, NSW).

### Instrumentation

The Shimadzu LCMS 8050 system included an autosampler, solvent pumps, a column oven chamber, and a triple quadrupole mass spectrometer (Shimadzu Australia, Rydalmere NSW). Analytes were separated during the LC phase using a C18 Poroshell 120 SB 2.1 × 50 mm 2.7 μm Column (Agilent Technologies Mugrave VIC) with a matching guard column.

#### Sample Preparation

Following thawing at room temperature from −80°C, 100 μl of sample was added to the pass-through plate, 390 μl of internal standard working solution (MFA), and 110 μl ACN were added and aspirated several times to mix. The samples were drawn through the plate into the wells of a 96-well round bottom plate by −15 psi negative pressure for 5 min. This plate was capped and placed in the autosampler, which was maintained at 4°C. Calibration standards were included on every plate.

#### LCMS Method

The mobile phases consisted of 0.1% v/v aqueous formic acid (aqueous) and 100% ACN (organic), and needle wash was 100% ACN. A total flow rate of 0.35 ml/min was maintained throughout the analysis with a linear gradient from 5.0% organic to 95% organic over 4.0 min. The conditions were returned to the starting conditions over another 1 min and held at this point for a further 3 min to allow re-equilibration. The injection volume was 5 μl.

The analytical and guard columns were maintained at 50°C. Nebulizing gas flow was 1.5 L/min, heating gas was 12 L/min, and drying gas was 8 L/min of nitrogen. The interface and DL temperatures were 300°C with the heating block held at 400°C. The collision gas was argon, with detection in the third quadrupole in multiple reaction monitoring (MRM) mode.

Positive ionization was utilized for the internal standard MFA with the precursor ion set at 297.10 m/z, and product ions at 279.10 and 244.05 m/z having collision energies (CE) of −13.0 and −25.0 eV, respectively. Negative ionization was employed for carprofen with the precursor ion set at 272.10 m/z, and product ions of 228.20 and 226.10 m/z having CE of 14.0 and 13.0 eV, respectively (31).

Validation of the method was carried out with intra-assay variability <10% CV and inter-assay variability was <16% for concentrations between 50 and 0.125 ng/ml, inter-assay variability for 0.0625, 0.0313, and 0.0156 ng/ml was 20.7, 38.2, and 51.9% CV, respectively. The signal-to-noise ratio at 0.0156 ng/ml was >10, therefore, the limit of detection was deemed to be below this level. A lower limit of quantification is not reported, as recently recommended (32).

#### ELISA

Duplicate samples were processed using commercial ELISA kits (Cayman Chemical; Prostaglandin E Metabolite ELISA Kit Item No. 514531 and Thromboxane B_2_ ELISA Kit Item No. 501020). Thromboxane concentrations were measured in serum samples as indicated in the instructions, without sample purification. A prostaglandin metabolite was measured in a selection of tissue cage fluid samples. Because native prostaglandin-E_2_ is unstable and rapidly metabolized *in vivo*, with an extensive first pass effect through the lungs, measurement of the metabolite produces a more reliable measurement of the PGE2 generated (30). The commercial method was modified by exchanging an ACN precipitation in place of ethyl acetate extraction. Samples were evaporated to dryness in a centrifugal evaporator (Environmental Speedvac Savant, USA) on a medium setting (42°C) for approximately 1.5 h before being resuspended in the ELISA buffer and derivatised with the supplied carbonate buffer overnight to produce a single stable compound for analysis. The derivatised samples were used directly in the ELISA without acidification.

The intra-assay precision reported in the PGEM kit manual was 8.1–23.7% CV, and the inter-assay precision was 7.2–123% CV. The reported interference from non-PGE molecules was 0.08% or less (30). For the thromboxane kit, the intra-assay precision is reported as 8.2–15.3% CV, the inter-assay precision is 9.9–12.9% CV, with interference from non-thromboxane molecules of 0.8% or less (33). Further validation was not performed because of the cost and material constraints.

### Statistical and Data Analysis Methods

Carprofen concentration data were modeled in Monolix (Lixoft, Antony France) utilizing a custom-built, population pharmacokinetic model which was fitted to the intravenous plasma data first then expanded to include the tissue cage data. Covariate data for sheep, period, cage side, carrageenan in cage, and carrageenan in sheep were included in the data set as discrete variables. Cage size (cm) was included as a continuous covariate variable.

In total, two thousand iterations were run to achieve convergence, with automatic stopping disabled. Diagnostic plots of the Markov chain Monte Carlo chains were visually assessed for evidence that convergence had been achieved.

The individual and population predicted values were plotted with the raw data and were visually inspected for goodness-of-fit.

Pharmacodynamic data were analyzed in RStudio (34, 35). Initially, plots were explored for relationships between variables. Non-linear mixed effect models were created using the NLME package (36) and evaluated for goodness-of-fit visually by assessing quantile-quantile (Q-Q) plots, Akaike's Information Criterion (AIC) and coefficient of determination (R^2^) values [MuMin package (37, 38)].

## Results

### Pharmacokinetic Model

A 2-compartment model was found to be a reasonable fit for the plasma concentrations. This model was created in the absence of the tissue cage data and described the plasma pharmacokinetics of carprofen.

**Figure 1:**
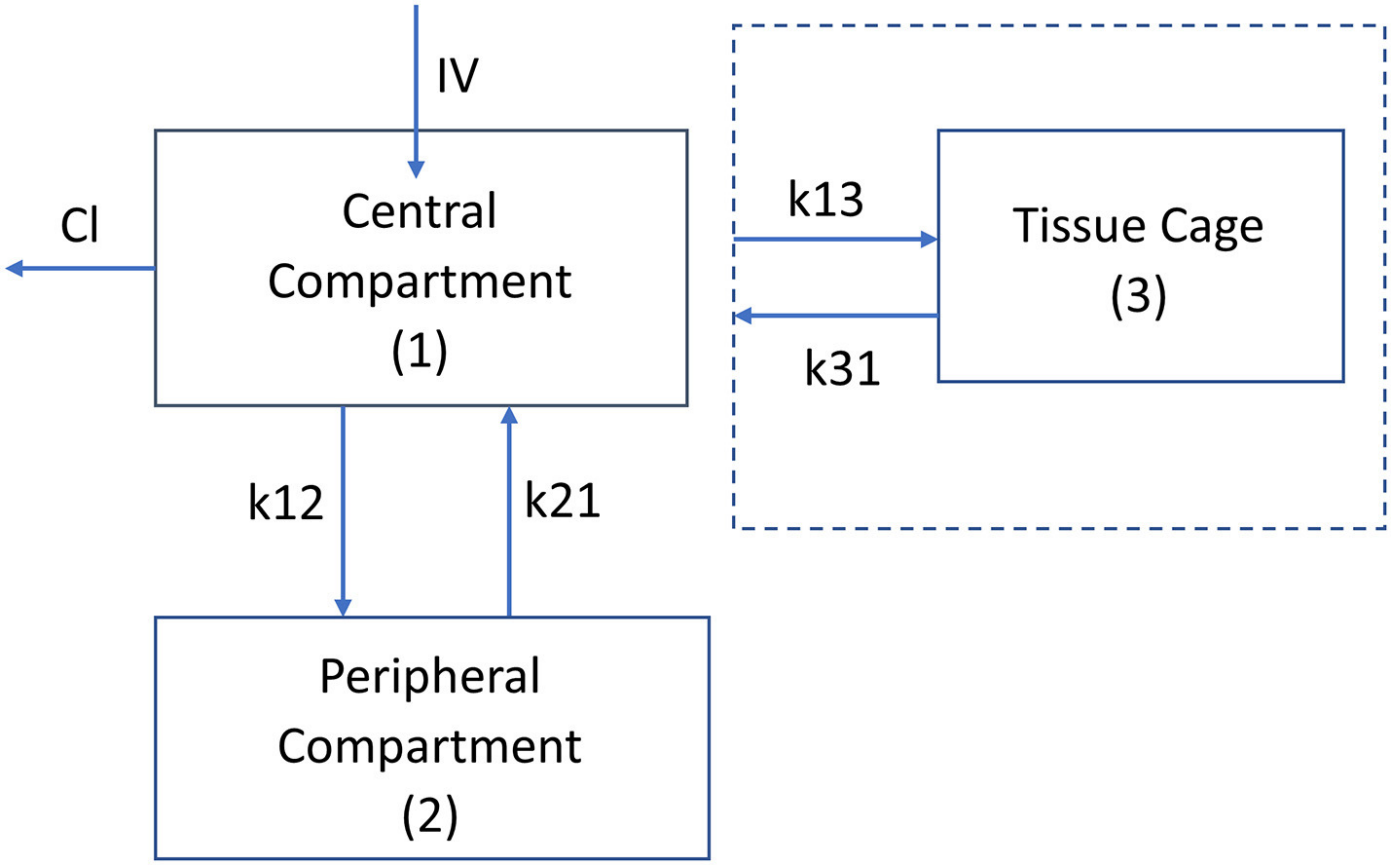
Schematic diagram of the pharmacokinetic model, plasma kinetics are model with a 2 compartment model with intravenous (instantaneous) administration into the central compartment (1). Clearance is from the central compartment. k12 and k21 are the rate constants (h^−1^) for the drug movement from the central compartment (1) to the peripheral compartment (2) and from the peripheral compartment to the central compartment, respectively. The kinetics of the tissue cage compartment are driven by the central compartment concentration with no change in the central compartment concentration. The drug's movement into and out of the cage is modeled by the rate constants k13 and k31, respectively. The volume of the tissue cage compartment is specified by the size of the tissue cage (Table 1).

A third compartment was added to the plasma model to represent the carprofen concentration in the tissue cages. The rates of influx and efflux (k_13_) are first order and are driven by the central compartment concentrations without altering the central compartment concentrations. The changes in the central compartment have already been accounted for in the “stand-alone” 2 compartment plasma model. This approach was taken because only a negligible proportion of carprofen drug would enter the tissue cages: this is similar to the approach taken by Sheiner et al. (39). A schematic depiction is displayed in Figure 1.

Table 3 shows the final model parameters generated and the relative standard error of those estimates. Of note is the relatively large change in k_21_ when carrageenan stimulates inflammation within the sheep, although this has a high degree of uncertainty. While the change in k_31_ for the right hand side has a low *p*-value (*p* = 0.0016), the magnitude of change is very small and the estimate is not precise (RSE 163%). Carrageenan administration was randomized between the left and right hand sides. Inflammation induced by carrageenan in the cage decreases the rate of drug movement both into and out of the cage, as shown by the change in k_31_ and k_13_. Cage size has a moderate effect on the rate of drug movement into and out of the cages, with a 25–30% change in the rate constants for each cm change in the cage length (*p* < 0.001). The half life for drug removal from the tissue cage is estimated to be 1.75 (1.08–3.36) h for a 3 cm cage to 15.4 (6.48–43.0) h for an 18 cm cage. The half-life for drugs entering the cage is estimated to be 6.29 (3.14–11.9) h for a 3 cm cage and 8.95 (3.03–29.6) h for an 18 cm cage. Table 5 displays the maxima, minima and median values of these rate constants.

**Table 3:** Coefficients for the model parameters estimated by Monolix.

**Fixed effects**	**Units**	**Maximum likelihood estimate**	**Relative standard error (%)**	**Confidence Interval 2.5%**	**Confidence Interval 97.5%**	***p-*value**
**POPULATION PARAMETERS ESTIMATION**						
Central volume	L/kg	0.0924	6.42	0.0846	0.101	
k12	h^−1^	0.121	7.91	0.0904	0.160	
Covariate for k12 for Carrageenan not in sheep	h^−1^	0^*^				
Covariate for k12 for Carrageenan in sheep	h^−1^	0.00114	9410	−0.324	0.327	0.992
k21	h^−1^	0.200	7.15	0.157	0.253	
Covariate for k21 for Carrageenan not in sheep	h^−1^	0^*^				
Covariate for k21 for Carrageenan in sheep	h^−1^	0.336	35.7	−0.0536	0.726	0.00507
Clearance	L/h.kg	0.00235	5.69	0.00192	0.00288	
k31	h^−1^	0.455	13.9	0.375	0.554	
Covariate for k31 for Cage Side (Left)	h^−1^	0^*^				
Covariate for k31 for Cage Side (Right)	h^−1^	−0.0701	163	−0.0863	−0.0539	0.0016
Covariate for k31 per cm Cage size	h^−1^	−0.147	8.14	−0.309	0.0153	<2.2e−16
Covariate for k31 for Carrageenan not in cage	h^−1^	0^*^				
Covariate for k31 for Carrageenan in cage	h^−1^	−0.104	130	−0.301	−0.0995	0.0331
k13	h^−1^	0.124	11.5	0.120	0.134	
Covariate for k13 for Cage Side (Left)	h^−1^	0^*^				
Covariate k13 for Cage Side (Right)	h^−1^	−0.024	377	−0.122	0.0743	0.0641
Covariate for k13 per cm Cage size	h^−1^	−0.0378	23.8	−0.0473	−0.0283	7.53e−13
Covariate for k13 for Carrageenan not in cage	h^−1^	0^*^				
Covariate for k13 for Carrageenan in cage	h^−1^	−0.184	58.3	−0.300	−0.0683	0.0045
**STANDARD DEVIATION OF THE RANDOM EFFECTS**						
Volume	L/kg	0.188	16.2			
Clearance	L/h.kg	0.448	15.8			
k31	h^−1^	0.522	10.8			
k13	h^−1^	0.482	7.43			
Error Model Parameters						
b1 (Plasma)		0.136	3.62			
b2 (Tissue Cage)		0.468	2.36			

*k12 is the rate constant for drug movement between the central compartment and the peripheral compartment, and k21 is the constant for drug movement from the peripheral compartment to the central compartment. k13 and k31 are the population rate constants for drug movement into and out of the tissue cages, respectively. These constants are modified by the covariates; the presence of carrageenan in the individual animal, with the reference being no carrageenan present for systemic pharmacokinetics. The tissue cage constants are modified by the cage length (size) in a continuous manner, i.e., k31 decreases by −0.147 for each centimetre of cage length. The presence of carrageenan in an individual cage and the side of the neck the cage is on also modifies the rate constants*.

* *The reference values are for the left hand side and there is no carrageenan in the cage*.

**Figure 2:**
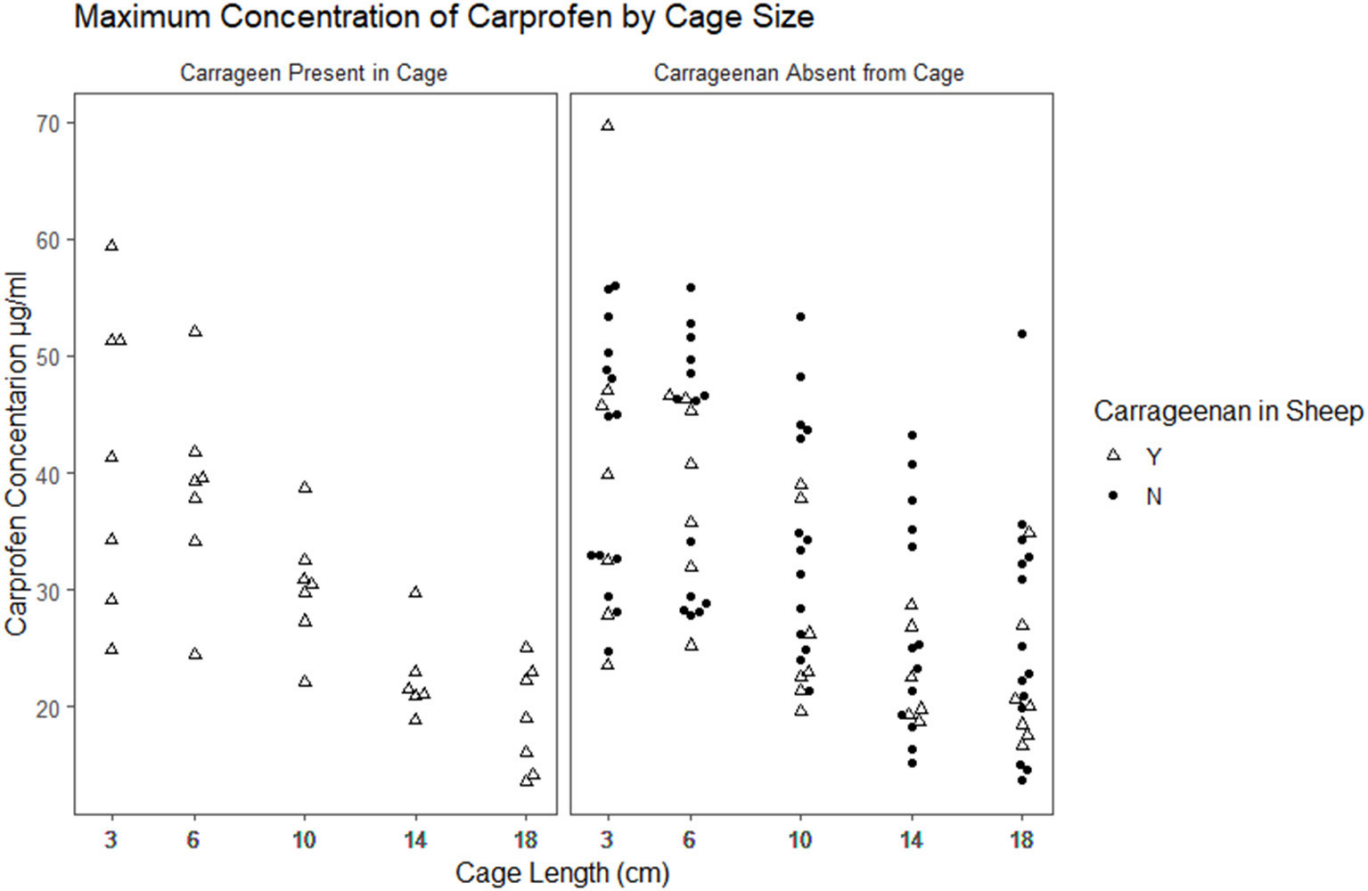
Scatterplot plot of the maximum observed concentration of carprofen by cage size and carrageenan presence within the cage.

**Figure 3:**
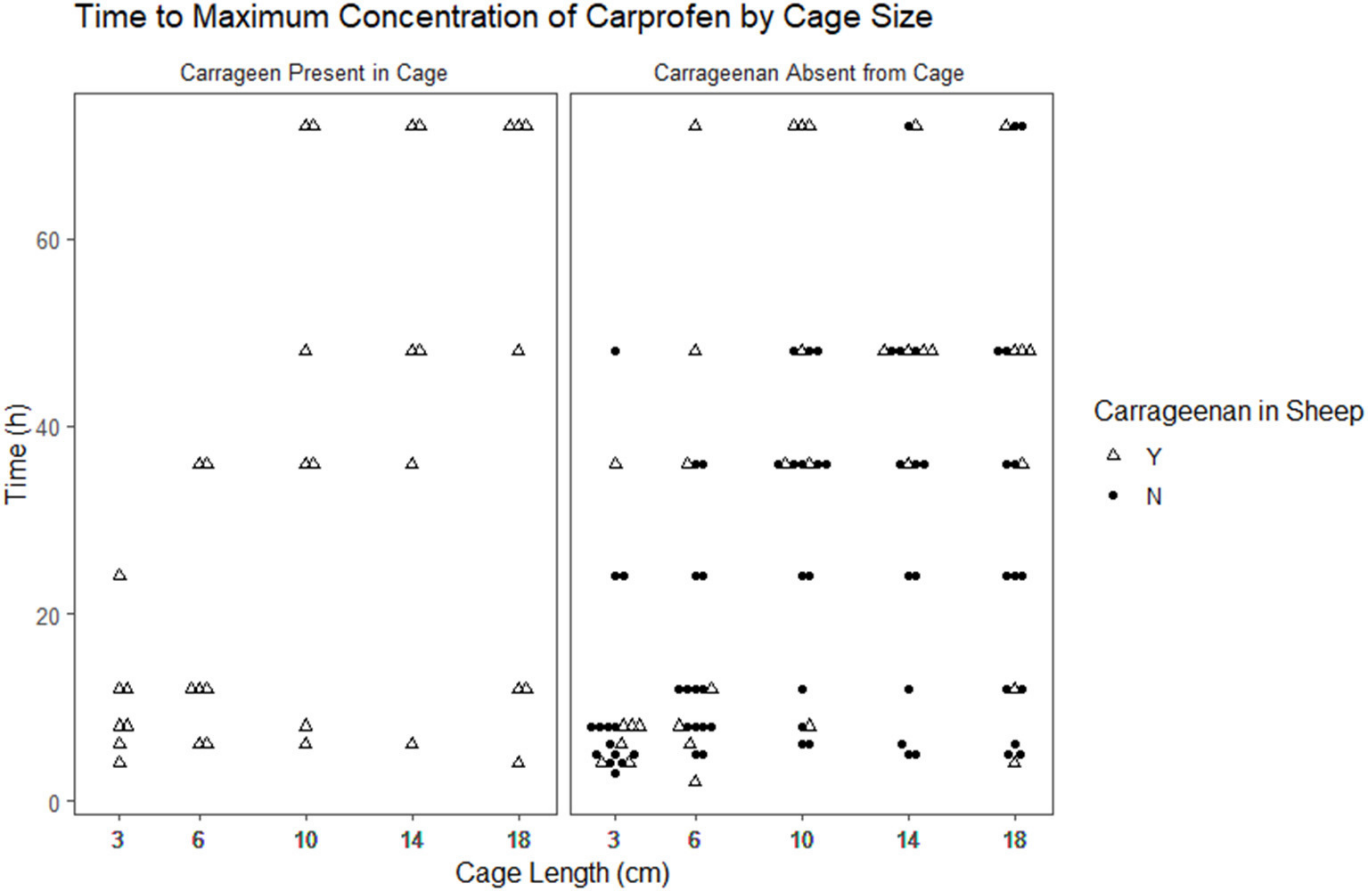
Scatterplot of the time to maximum concentration of carprofen by cage size and carrageenan presence within the cage.

The time to reach the maximum concentration of carprofen (Tmax) and the maximum concentration reached (Cmax) were extracted from the raw data for each cage in each period (Figures 2, 3). The median and range for these parameters by cage size are shown in Table 4. Tmax for the 3 cm cages had a median of 8 h, while the 14 cm cages had the longest time to maximum concentration with a 48 h median. The median Cmax observed was 43.10–21.57 μg/ml for the 3 to 18 cm cages, respectively.

**Table 4:** Median and range of observed time to maximum concentration (Tmax) in hours and the concentration of carprofen (Cmax) achieved in micrograms per millilitre for each tissue cage size implanted.

**Cage size**	**SA/V**	**Count**	**Tmax median**	**Tmax minimum**	**Tmax maximum**	**Cmax median**	**Cmax minimum**	**Cmax maximum**
3	0.57	28	8	3	48	41.45	23.54	69.62
6	0.28	28	12	2	72	39.82	24.50	55.82
10	0.17	28	36	6	72	31.85	19.66	53.38
14	0.12	25	48	5	72	25.01	15.12	43.16
18	0.09	28	36	4	72	23.57	13.54	51.92

**Table 5:** Maxima, minima, and median values of k13 and k31, the rate constants of drug movement into and out of the tissue cages, by cage size.

**Parameter**	**Cage size**	**Min k**	**Median k**	**Max k**	**Min t_**1/2**_**	**Median t_**1/2**_**	**Max t_**1/2**_**
k13	0	0.1330	0.1390	0.149	4.65	4.99	5.21
	3	0.0581	0.1060	0.221	3.14	6.54	11.90
	6	0.0530	0.1140	0.197	3.52	6.06	13.10
	10	0.0267	0.0862	0.158	4.39	8.03	26.00
	14	0.0248	0.0582	0.178	3.89	11.90	27.90
	18	0.0234	0.0694	0.229	3.03	9.98	29.60
k31	0	0.4880	0.5190	0.564	1.23	1.33	1.42
	3	0.2060	0.3860	0.644	1.08	1.80	3.36
	6	0.1000	0.1530	0.341	2.03	4.54	6.93
	10	0.0420	0.0703	0.173	4.00	9.86	16.50
	14	0.0284	0.0513	0.111	6.27	13.50	24.40
	18	0.0161	0.0400	0.107	6.48	17.30	43.00

*Based on individual mean predictions of the pharmacokinetic model. The half-life of absorption (k13) into the cage and elimination (k31) out of the cage is also displayed. This is calculated by the equation t1/2 = 0.693/k*.

Tables 6, 7 show the difference in estimated marginal means between the Tmax and Cmax for each cage size.

**Table 6:** The difference in estimated marginal means (contrast) between the maximum concentration of carprofen in the respective cage sizes.

**Contrast**	**Estimate**	**Confidence**	**Confidence**	***p*-value**
		**interval 2.5%**	**interval 97.5%**	
3–6	1.63	−3.42	6.68	0.524
3–10	9.60	4.55	14.60	<0.001
3–14	16.40	11.20	21.60	<0.001
3–18	17.90	12.80	22.90	<0.001
6–10	7.97	2.92	13.00	0.00219
6–14	14.80	9.62	20.00	<0.001
6–18	16.30	11.20	21.30	<0.001
10–14	6.84	1.65	12.00	0.0102
10–18	8.28	3.24	13.30	0.00148
14–18	1.44	−3.75	6.63	0.584

*The 95% CI and p-value are also displayed*.

**Table 7:** The difference in estimated marginal means (contrast) between the time to maximum concentration of carprofen in the respective cage sizes.

**Contrast**	**Estimate**	**Confidence**	**Confidence**	***p*-value**
		**interval 2.5%**	**interval 97.5%**	
3–6	−7.36	−17.80	3.04	0.164
3–10	−25.60	−36.00	−15.20	<0.001
3–14	−28.30	−39.00	−17.60	<0.001
3–18	−24.10	−34.50	−13.70	<0.001
6–10	−18.30	−28.70	−7.88	<0.001
6–14	−20.90	−31.60	−10.20	<0.001
6–18	−16.80	−27.20	−6.38	0.00177
10–14	−2.64	−13.30	8.07	0.627
10–18	1.50	−8.90	11.90	0.776
14–18	4.14	−6.57	14.80	0.446

*The 95% CI and p-value are also displayed*.

### Pharmacodynamic Results

A total of 279 PGEM results were available for analysis and, of these, 74 results were from cages without carrageenan. This imbalance was intentional due to the expectation that non-inflamed cages would have PGEM concentrations below the level of detection, all samples analyzed were above the LOD. All the samples were taken from animals that received carprofen, therefore, the expected PGEM concentration without carprofen is not known and the 50% inhibitory concentration (IC50) cannot be calculated. Overall, the PGEM concentrations increased from time zero to 72 h after carprofen administration with a high degree of variability.

The prostaglandin E2 metabolite results were modeled with a linear mixed effects model. PGEM was log transformed, and cage size was analyzed as a discrete covariate with 5 levels. Individual sheep was included as a random variable. The concentration of carprofen in the cage was not included in the final model as it is collinear with time.

The final model was


$$\begin{array}{l} \log_{10}\;\left(PGEM\right)\;\sim \;\left(\beta 0,\;\gamma sheep\right)\;+\;\beta 1\;.Time+\;\beta 2.Cage\;Size\;\\ +\;\beta 3.Time.Cage\;Size\;+\;N\left(0,\sigma \right) \end{array}$$

where the logarithm of PGEM concentration is predicted by time, cage size, and their first order interaction with individual sheep is included as random effects. β_0_ is the estimated population intercept and γ*sheep* is the variance in intercept for the individual subject. σ is the SD of the unexplained variability.

The model explains some of the variation seen with the marginal and conditional *r*^2^ of 0.27 and 0.42, respectively.

The coefficients of the fixed effects and their interactions are displayed in Table 8. Time is a significant predictor of PGEM concentration in this model. Only the 14 cm level of the cage size covariate differed significantly from the 3 cm cage reference. A significant interaction occurs (*p* = 0.0029) between the 6 cm cage and time in our dataset.

**Table 8:** Table of coefficients for the fixed effects and interactions for the PGEM mixed effect model.

**Term**	**Estimate**	***p*-value**	**Confidence**	**Confidence**
			**interval 2.5%**	**interval 97.5%**
(Intercept)	1.55000	<0.001	1.37000	1.720000
Time	0.00731	<0.001	0.00317	0.011500
CageSizeFac6	0.01580	0.839	−0.13700	0.169000
CageSizeFac10	0.06640	0.395	−0.08710	0.220000
CageSizeFac14	0.16900	0.0385	0.00899	0.329000
CageSizeFac18	0.06440	0.423	−0.09360	0.223000
Time:CageSizeFac6	0.00800	0.00295	0.00275	0.013200
Time:CageSizeFac10	−0.00160	0.551	−0.00687	0.003670
Time:CageSizeFac14	−0.00416	0.161	−0.01000	0.001670
Time:CageSizeFac18	−0.00501	0.0693	−0.01040	0.000398

*The 95% CI and p-value is displayed for each covariate*.

**Figure 4:**
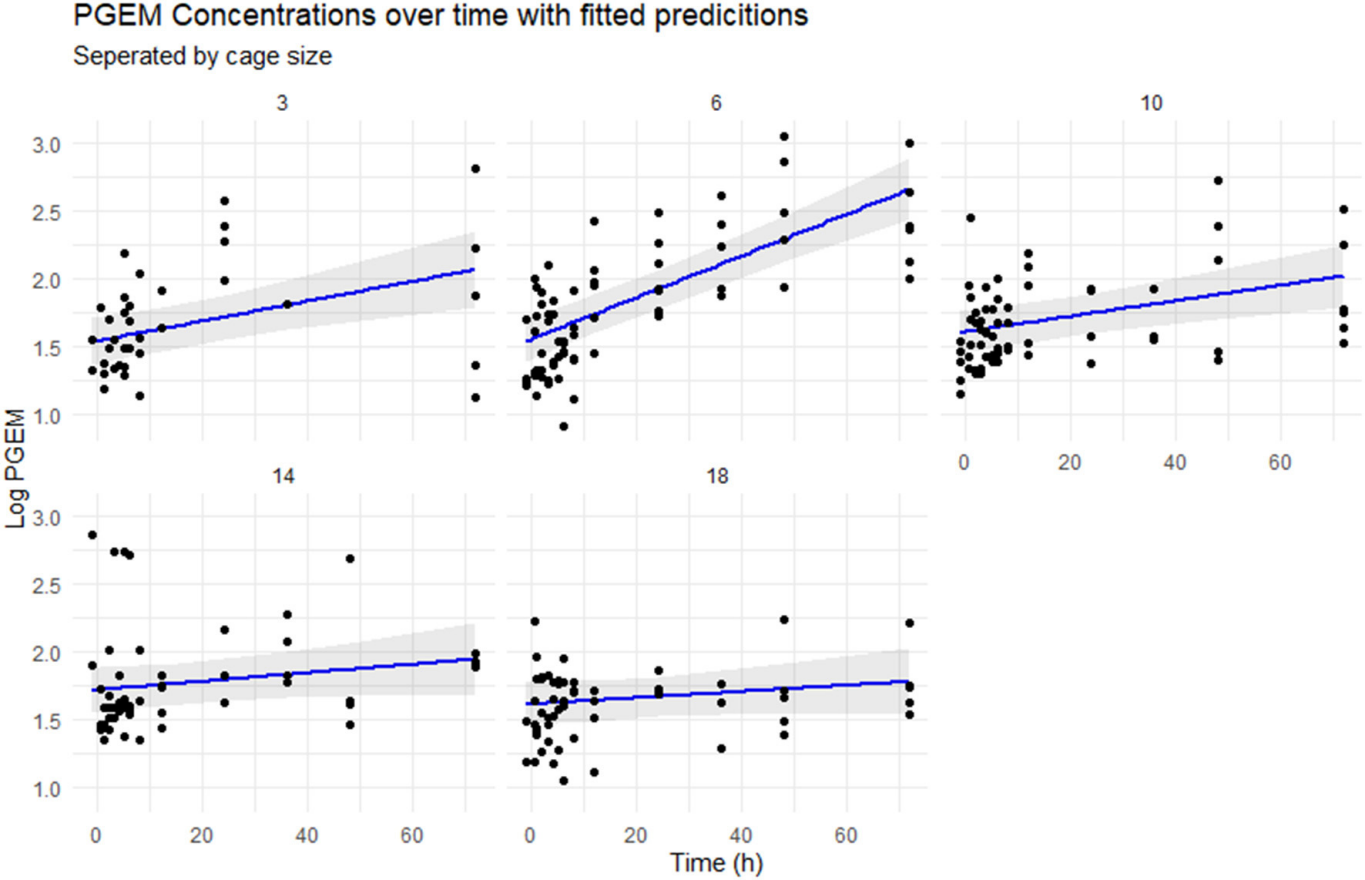
Scatterplot of the logarithmically transformed PGEM concentrations over time, separated by cage size. The blue line represents the concentrations predicted by the mixed effect model, with 95% CIs in gray.

The fitted linear model (Figure 4) shows a rapid increase in PGEM concentrations in the 6 cm cages compared with the other cage sizes, with a predicted concentration of 0.75–1 logarithm (2 vs. 2.75) higher than the other cages. There is significant variability in the results within timepoints, with the 3 cm cage having approximately 1.5 logarithms spread at the 72 h timepoint.

## Discussion

The plasma pharmacokinetic parameters are similar to those reported by Welsh et al. (40). The terminal plasma half-life reported by Welsh was 33.7 h for 4 mg/kg and 26.1 h for 0.7 mg/kg, the terminal plasma half-life estimated in this study is 27.2 h. The volume of distribution reported by Welsh was 117.3 and 92.7 ml/kg with our model point estimate for the population being 92.4 ml/kg. A small degree of enterohepatic recirculation was suspected at 4 mg/kg by Welsh based on visual inspection of the raw data plots and this was also seen in our data, although it was not included in the PK model.

As previously reported, there is some evidence for carrageenan-induced inflammation slowing the movement of drugs into and out of the tissue cage, thus, prolonging their effect. This effect was highly uncertain, as evidenced by the high relative standard errors. The change in plasma kinetics when carrageenan is present in the individual animal is of note. The rate constant of drug return to the central compartment (k_21_) more than doubled at a population level when carrageenan was present in the sheep, with a large residual uncertainty in this estimate. This change in plasma kinetics in the presence of local inflammation is an important point as previous models have included carrageenan in all sampling periods and compared the pharmacokinetics and pharmacodynamics between inflamed and non-inflamed cages in the same individual (21, 22). The values from the non-inflamed cages in these studies may not accurately reflect PK in the true absence of inflammation, as our findings showed changes in the systemic pharmacokinetics in cases with tissue cage localized inflammation. It is important to note that the cumulative amount of carrageenan introduced in this model is higher than in other published models. All five cages on one side had carrageenan introduced, as opposed to most other studies where carrageenan is introduced into only one cage within the animal per sampling period. The model presented in this article could be criticized as all cages received 1 ml of carrageenan regardless of cage volume. This may lead to unequal degrees of inflammation between the cages as the smaller cages would have a higher concentration of carrageenan. The degree of inflammation induced was not measured.

We detected a clear negative effect on the rate of drug movement into and out of peripherally implanted tissue cages based on the cage length. The resulting concentration time curves are visually different, with key parameters Tmax and Cmax varying with cage size. Tmax occurred later in larger cages, with a 6-fold change between the earliest median Tmax and the longest. Cmax was lower in the larger cages, with the median Cmax in the 18 cm approximately half of the Cmax observed in the 3 cm cages. This result is expected, as the Fick's law predicts that the diffusion is proportional to the concentration gradient and the surface area, i.e., the amount of drug entering or exiting a cage is limited by the diffusable surface area (the total area of the fenestrations). If we assume that the drug is equally dispersed within the cage, then the concentration is a function of the surface area to volume ratio of the cage. Bengtesson et al. (41) describe an equation based on Fick's law to model tissue cage concentrations. It includes the free concentration of drug in the cage and serum at any given time, the surface area to volume ratio of the cage, and a constant for the permeability of the tissue between the blood vessels and the cage. They also assume that the amount of drug in the tissue is so small compared to the serum that it will not affect the serum concentrations, as is the case for the PK model we describe in this article. In this study, we measured total carprofen with the implicit assumption that the unbound proportion would remain constant. Other studies have examined the protein concentrations of tissue cage fluid during sampling periods and found them to be relatively stable (3) which supports our assumption.

Carprofen is a racemic drug with a single chiral center, pharmacokinetic differences between the two enantiomers have been shown in sheep, horses, and dogs but no evidence of *in vivo* chiral conversion was found (7, 19, 42). The length of the tissue cage was used as a surrogate measure for SA/V in the pharmacokinetic model as the model failed to converge with SA/V or logarithmically transformed SA/V. However, the work by Van Etta (5, 6) demonstrated that SA/V was the parameter of interest, not the physical size of the tissue cage. Nevertheless, because the diffusable surface area was held constant between cages of different lengths in this study, the length and SA/V are directly related and colinearity occurs, so these effects under this design have poor identifiability. Ideally, the model would include variation in both surface area and volume.

It appeared from our data that carprofen suppressed the inflammation in the early stages of the experiment, as expected. This is in agreement with Cheng (21) with substantial suppression of PGE by carprofen for 32 h. While our model did not explicitly include the concentration of carprofen as an explanatory variable, carprofen concentration was highly correlated with time by design, so it would add no meaningful information.

The primary aim of this study was to evaluate the effect of SA/V and inflammation on carprofen's PK and PKPD results estimated using a tissue cage model, where we had hypothesized that as the volume of the cage increases relative to the surface area, changes would occur to the estimated pharmacokinetic and pharmacodynamic parameters. Our results showed that different SA/V ratios changed the observed PK and PKPD of carprofen. It is now clear that the SA/V ratio of subcutaneously implanted tissue cages markedly affects derived pharmacokinetic parameters, with the highest median Cmax double the lowest median observation and the longest median Tmax five times greater than the shortest median observation. Our findings give weak evidence that dependent pharmacodynamic parameters may also be influenced by the SA/V ratio.

Understanding the relationship between the SA/V ratio and observed PK may allow results from other studies where the SA/V is known to be extrapolated and compared in a meta-analysis (43). If a target body tissue or compartment, such as a joint space, had a known SA/V ratio and permeability constant, then, dosage regimens could be accurately simulated to provide target concentrations of drug at the site of interest. It is this linking of tissue cage data to real biological spaces that would allow the true potential of tissue cage models to be utilized.

## Data Availability Statement

The raw data supporting the conclusions of this article will be made available by the authors, without undue reservation.

## Ethics Statement

The animal study was reviewed and approved by Faculty of Veterinary and Agricultural Science Animal Ethics Committee, University of Melbourne.

## Author Contributions

RM, TW, and AW contributed to the planning, design, execution, and analysis of the study. All authors contributed to the article and approved the submitted version.

## Funding

RM was supported by the Australian Government Research Training Program Scholarship during this work.

## Conflict of Interest

The authors declare that the research was conducted in the absence of any commercial or financial relationships that could be construed as a potential conflict of interest.

## Publisher's Note

All claims expressed in this article are solely those of the authors and do not necessarily represent those of their affiliated organizations, or those of the publisher, the editors and the reviewers. Any product that may be evaluated in this article, or claim that may be made by its manufacturer, is not guaranteed or endorsed by the publisher.
